# Road traffic accidents in Ghana: contributing factors and economic consequences

**DOI:** 10.4314/gmj.v54i3.1

**Published:** 2020-09

**Authors:** Paa Kwesi Blankson, Margaret Lartey

**Affiliations:** Oral and Maxillofacial Surgery Unit, Korle-Bu Teaching Hospital p.blankson@kbth.gov.gh; Department of Medicine & Therapeutics, University of Ghana Medical School mlartey@ug.edu.gh

The impact of road traffic injuries could be enormous, affecting societies and individuals in different facets. Despite the prominence of Covid-19 disease in the current public health space, road crashes remain an important contributor to mortality. It is estimated that some 1.4 million people die from road crashes globally, with most of these being the youth, and people from developing countries.^1^ In Ghana, 72 persons out of every 100 000 population, suffered from grievous bodily injury, and close to 8 of the same population died from Road Traffic Accidents (RTAs) over the past decade. More than 60% of road traffic fatalities occured in children and young persons under 35 years of age.^2^ Aside the mortality and morbidity associated with the road crashes, Ghanaian households spend an average of US$ 1687.65 in direct and indirect cost on severe injuries associated with road crashes, while many suffer considerable degrees of psychological distress.^3^

The burden of road crashes in Ghana require a conscientious and multi-sectorial approach to reduce its occurrence and impact, while policies need strengthening and enforcing at all levels. The impact of RTAs may be mitigated by efficient emergency systems as well as policies which support care of victims. However, at the primary level, implementation of traffic rules and regulations is key, as discussions on road and vehicle safety are broadened. Road safety should be of concern to all stakeholders, as both motorists and pedestrians are at risk of the hazards of road crashes.

At the Korle-Bu Teaching Hospital in Accra, for instance, road traffic accidents accounted for 62% of deaths at the casualty unit of the emergency department. Interestingly, among the deaths caused by road crashes, 50% occurred in pedestrians, 31% in passengers and 18.7% occurred in motorists. It is worthy of note that among persons injured from road traffic crashes in this same study, 26% were related to motorbike accidents.^4^ Road safety education is also a crucial element in the prevention strategies for road traffic accidents. At one local maxillofacial centre, majority of the injuries encountered (55%) were from road traffic accidents.^5^ Management of these cases, often with limited resources, brings to the fore another dimension of concern for many healthcare professionals- the burden on the healthcare system and the families of the patients.

Despite the disease burden, there seem to be a relative dearth of research on road crashes in Ghana. In this issue of the Ghana Medical Journal, Poku et al report some instructive findings on factors associated with road crashes from drivers' perspective in the Kintampo North Municipality. Their findings indeed provide useful pointers to areas requiring some actions.

Providing evidence through research on the burden, regional distribution, health system factors, cost-effective interventions and policy effectiveness should stimulate various stakeholders to take up the challenge and help reduce this preventable and avoidable catastrophe.
